# Updated Review on Ozone Therapy in Pain Medicine

**DOI:** 10.3389/fphys.2022.840623

**Published:** 2022-02-23

**Authors:** Francisco Javier Hidalgo-Tallón, Luis Miguel Torres-Morera, Jose Baeza-Noci, Maria Dolores Carrillo-Izquierdo, Rosa Pinto-Bonilla

**Affiliations:** ^1^Institute of Neurosciences, University of Granada, Granada, Spain; ^2^Department of Anesthesia, Resuscitation, and Pain Treatment Service, Hospital Puerta del Mar, Cadiz, Spain; ^3^Department of Embryology and Human Anatomy, School of Medicine, University of Valencia, Valencia, Spain; ^4^School of Nursing, Catholic University of Murcia (UCAM), Murcia, Spain

**Keywords:** ozone therapy, ozone injections, medical ozone, pain medicine, chronic pain

## Abstract

The use of medical ozone in the treatment of chronic pain is progressively expanding in Spain and today it is used both in public and private medical centers. However, there is a great lack of knowledge about this technology not only in primary care but also in medical specialties. Although its biochemical bases are well determined and there are various systematic reviews and meta-analyses in the literature that justify its use in pain medicine, some professionals still are prejudiced against it. The evidence level of using medical ozone according SIGN (Scotish Intercollegiate Guideline Network) criteria is similar or superior to most of the techniques used in a Pain Unit. In this paper, we have done a review on ozone therapy in pain medicine, compiling the evidence published about it.

## Introduction

Ozone therapy is the use of medical ozone as a therapeutic substance in pathologies with chronic hypoxia, inflammation, and redox imbalance in which ozone has proven to be effective (Baeza et al., 2015). Medical ozone is a mixture of oxygen and ozone obtained from medical oxygen by using a medical device – a medical ozone generator approved by a Notified Body according to the European Directive 93/42 and national regulations (Spanish RD 1591/2009). Medical ozone generators for parenteral use are classified in heading IIB of the classification of medical devices in EU regulations. Ozone therapy in medicine is a growing reality, and there are more and more professionals using medical ozone as a therapeutic tool for different diseases related to chronic oxidative stress and inflammation, including chronic pain.

In addition to health professional associations (such as the Spanish Ozone Therapy Association—SEOT or the World Federation of Ozone Therapy—WFOT) that try to unify criteria and develop protocols of treatment, as well as training health professionals in the use of this substance, the Catholic University of Murcia has taken the initiative by creating a chair of Ozone Therapy and Chronic Pain to better promote training and researching inside the Spanish universities network.

In Portugal and Greece, ozone therapy has a specific regulation and is used in public and private centers. In the rest of the European Union, it is used thanks to the legal recognition of medical ozone generators. In 2011, the Spanish Ministry of Health included the ozone therapy in the portfolio of services of the pain units (Palanca-Sánchez et al., 2011), so it is necessary that doctors who are experts in pain management know the rationale of medical ozone therapy and how it works, both locally and systemically.

Ozone is a molecule composed of three oxygen atoms (O_3_) instead of the two oxygen atoms found in the oxygen molecule (O_2_). Ozone has a half-life of 40 min at 20°C (Baeza et al., 2015); for this reason, it cannot be stored and must be produced “*in situ*” for each application.

Medical ozone applications date back to the beginning of the last century. The papers compiled in this publication have been published in the last 25 years and the oldest one have been included for historic reasons. Dr. Kellogg, in his book on diphtheria (1881) already mentioned ozone as a disinfectant, and in 1898 Drs Thauerkauf and Luth founded in Berlin the “Institute for oxygen therapy,” carrying out the first trials with animals. In 1911, the book “A Working Manual of High-Frequency Currents”, published by Dr. Noble Eberhart, head of the department of physiological therapeutics at Loyola University, described the use of medical ozone in the treatment of diseases such as tuberculosis, anemia, asthma, bronchitis, hay fever, diabetes, etc. (Pressman, 2021). But despite the successes achieved at the beginning of the last century, the ozone generating machines lacked precision and were quite fragile as they used a lot of glass tubes.

**Figure 1:**
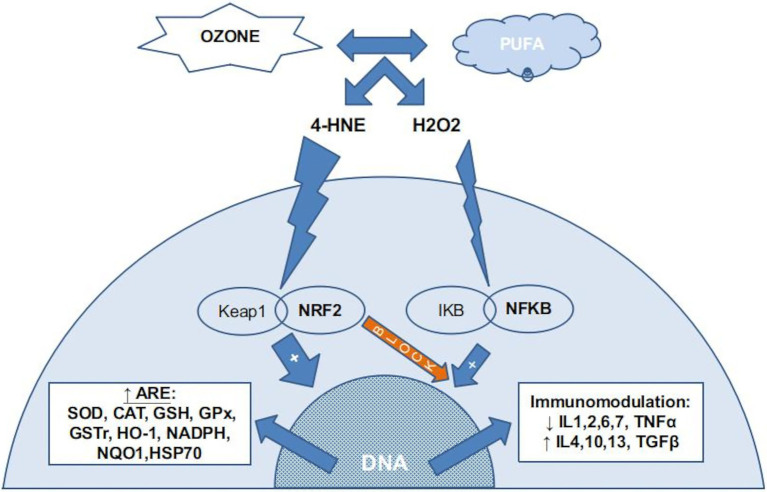
The chemical reaction between ozone and PUFA (poli-unsaturated fatty acids) in the insterstitial water (or plasma) produce several ROS, mainly H_2_O_2_, and several LOPs, mainly 4-HNE (4-hydroxynonenal). Almost all H_2_O_2_ is kidnaped by erythrocytes (not reflected in this figure) if present. In nucleated cells, LOPs activates the NRF2 pathway, inducing the production of ARE (antioxidant response elements) and blocking the NFKB pathway. A small amount of H_2_O_2_ stimulates the NFKB pahtway, usually balanced with the NRF2 blocking action, producing a inmmunomodulation.

When applying this therapy, we are really inducing a controlled and harmless “micro-oxidation” that will produce a modulation of the cellular antioxidant system and the inflammation system. Ozone reacts with interstitial fluids producing hydrogen peroxide (H_2_O_2_), aldehydes, and lipid oxidation products (LOPs). These substances induce activation of the nuclear factor erythroid 2-related factor 2 (NRF2) pathway that will induce an increase in antioxidant systems (ARE), such as superoxide dismutase (SOD), catalase (CAT), reduced glutathione (GSH), glutathione peroxidase (GSH-Px), glutathione s-tranferase (GSTr), hem-oxygenase-1 (HO-1), reduced nicotinamide adenine dinucleotide phosphate (NADPH), NADPH quinone oxidoreductase 1 (NQO1) and heat shock protein-70 (HSP70; Baeza et al., 2015). This NRF2 activation (Figure 1) induces a decrease in the nuclear factor kappa beta (NFKβ) pathway activity, inducing an anti-inflammatory effect [decrease of interleukynes 1,2,6,7 and tumor necrosis factor alpha (TNFα) and increase of interlukynes 4, 10, 13 and transforming growing factor beta (TGFβ)] In the injected tissues, medical ozone inactivates proteolytic enzymes through the inhibition of the NFKβ pathway. At the same time, there is a proliferation of fibroblasts and chondrocytes, favoring cartilaginous regeneration (Fernández-Cuadros et al., 2020).

Several authors have published preclinical studies on the effects of medical ozone on living organisms, being able to demonstrate beneficial effects on the ability to modulate the redox balance, the cellular inflammation status, and the adaptation to ischemia/reperfusion processes (Barber et al., 1998; Peralta et al., 2000; Ajamieh et al., 2003, 2004, 2005).

From a clinical point of view, ozone therapy presents multiple medical-surgical applications, all of them related to the germicidal capacity of ozone, chronic ischemic and inflammation processes, and imbalance of the cellular redox status. Several manuals collect the experience and scientific work carried out to date by different research groups, mainly Italians, Germans, Russians, and Cubans (Bocci, 2002; Menéndez et al., 2008).

The forms of application of medical ozone are basically three: topical, infiltrative, and systemic (Baeza et al., 2015). Topical applications take advantage of the germicidal power of ozone and its positive effect on the healing processes; it is usually applied directly, with the use of zip-lock bags, or by using ozonated water or ozonated oils. Infiltrated ozone at concentrations between 4 and 30 μg/ml is useful for treating musculoskeletal diseases such as arthritis, tendonitis, myositis, fasciitis, neuritis, or myofascial pain. Systemic ozone therapy consists of the administration of the mixture of gasses mainly through two routes: the indirect intravenous way (also known as autohemotherapy) and the rectal insufflation. Indirect intravenous administration consists of extracting a determined blood quantity that, within a closed circuit, is put into contact with the gas, which will dissolve and react in a few seconds, and immediately reinfused. Certified (EU marked) medical devices should be used for this procedure. Rectal insufflation consists of administering a gas enema with a probe into the rectum, where it reacts with rectal mucus and generates peroxides. These are absorbed by the mucosa, reaching the hemorrhoidal plexus and the general venous blood circulation system like any other rectal treatment. The probes must be made of silicone or other ozone-resistant plastic (OzoneSolution, 2021).

## Infiltrations With Medical Ozone (O_2_/O_3_)

### Generalities

The use of O_2_/O_3_ infiltrations to treat musculoskeletal pathology is increasingly widespreading. Verga (1989) was the first to describe ozone intramuscular applications at the paravertebral level and trigger points in patients with chronic low back pain. Later, in the 90s, this procedure was also used to treat acute and chronic polyarthritis (hip, knee, sacroiliac joint, and interphalangeal), tendinitis, epicondylitis, carpal tunnel syndrome, and myofascial pain (Ajamieh et al., 2005).

Despite its frequent use, the level of evidence in many of the mentioned indications, but for the treatment of lumbar disk herniation and knee osteoarthritis, is low, possibly because it has been mainly used for years in private practice where randomized clinical trials are difficult to carry out. Carmona (2005) published in 2005 a systematic review on the efficacy of ozone therapy in rheumatic diseases, concluding that there were no quality clinical trials, that most of the works were published in low-impact journals, and that the methodology between the different studies is highly variable. However, this situation is changing, as more countries are using medical ozone in public centers and universities.

### Therapeutic Properties and Mechanisms of Action of Injected Ozone

When we infiltrate the oxygen/ozone mixture, we are infiltrating a highly oxidizing gas, with a good tissue diffusion capacity. Apart from the general biochemical effect described in the Introduction section, several authors have described the anti-inflammatory, analgesic, and anti-edema properties of injected medical ozone, and propose that the oxidation of the algogenic receptors would inhibit the pain signal and it would activate the antinociceptive system (Re et al., 2010). This fact has been checked in one preclinical study from Fuccio et al. (2009) by inducing sciatic damage in mice, He confirmed the corticofrontal activation of genes of caspase 1, 8, and 12 (pro-inflammatory, pro-apoptotic, and responsible for allodynia) caused by the injury; this expression was normalized with a single peripheral injection of O_2_/O_3_ around the damaged area, which also reduced mechanical allodynia.

These properties would favor a muscle relaxant effect, as well as improved mobility of the treated area that can be observed clinically (Siemsen, 1995). This fact is very important in muscle recovery with O_2_/O_3_ injections. Balkanyi (1989) has described the utility of ozone therapy in the treatment of painful muscle hypertonia, highlighting the tremendous muscle relaxant effect that is produced.

Regarding the nucleus pulposus of the intervertebral disk, several authors have described that ROS will react with amino acids and carbohydrates of proteoglycans and collagen I and II that make up the matrix, giving a lead to a process of “mummification” which would shrink the disk, reducing the compression (Hawkins and Davies, 1996; Bocci et al., 2001; Leonardi et al., 2001). Iliakis and his group studied the histological changes of the nucleus pulposus matrix after treatment with an ozone intradiscal injection at a concentration of 27 μg/ml in rabbits and humans (resected disk specimens using microdiscectomy after the failure of the ozone treatment). Five weeks after the injection there were no signs of perilesional chondrocytic hyperplasia or inflammatory infiltrates typically observed in herniated disks biopsies; there was a fibrous tissue of less volume (“disk mummified”), which supposes a decrease in compression on the nerve root, a decrease in venous ecstasy, an improvement in local circulation, greater oxygenation and less pain, given the great sensitivity to hypoxia of nerve roots (Iliakis et al., 2001). Similar findings in pigs at different ozone concentrations were reported by Muto (2004).

### Dosage

Regarding dosage, standardized protocols are lacking. Most authors relate the amount of the gas mixture to the extension of the injury or to the size of the joint cavity to be infiltrated. Generally, the amounts of gas range between 5 and 15 ml, and ozone concentrations range from 4 to 30 μg/ml. The number of infiltration sessions usually ranges from 3 to 10 (usually one or two per week) depending on the specific evolution of each case. About intradiscal injection, 2 ml for cervical and 5 for ml is the most used amount. If a patient does not respond to treatment after two or three interventions (once every 2 weeks) it is considered a failure. Torres et al. (2009) in a 2009 publication, after having used different concentrations with the same clinical protocol, observed more improvements when intradiscal O_2_/O_3_ is infiltrated at 50 μg/ml compared to lower concentrations (25 and 30 μg/ml). Nevertheless, we recommend not reaching 50 μg/ml due to the risk of iatrogenic injuries on the ring that have been detected experimentally in pigs (Muto, 2004).

This lack of standardization in the treatment protocols makes difficult to compare results when performing a systematic review, not allowing to get high quality conclusions or recommendations.

### Ozone Therapy in Knee Pathology

One of the historic references was done in 1989 by Riva (1989), that registered 156 patients with knee joint pathology (post-traumatic arthritis, knee osteoathritis with mild deformity, and knee osteoathritis with severe deformity) obtaining good results that were especially positive when there were no severe bone deformities. The treatment consisted of intra-articular and periarticular infiltrations of 10 ml of medical ozone at a concentration of 20 μg/ml.

Later, In Cuba, a prospective study was carried out in 1997 to evaluate the efficacy and safety of medical ozone injections in 126 patients with knee osteoarthritis; usually, three or four injections were needed to obtain positive results, and only 14 patients received more than five sessions. 71.4% of the patients had a good result, in 10.3% the result was fair, and in 18.3% the result was bad. The main complication was pain during infiltration. It was highlighted the economic savings due to the low cost of the infiltrations and the lesser need for anti-inflammatory drugs (Escarpanter et al., 1997). Several authors have also published their experience in case series (Delgado and Quesada, 2005; Huanqui et al., 2006) and even proposed a treatment guide for based on their personal experience (Gheza and Bissolotti, 2003).

Moretti et al. (2004a) worked on the early knee osteoarthritis, comparing intraarticular injected O_2_/O_3_ with intraarticular hyaluronic acid, concluding that although there were no statistically significant differences in efficacy, ozone could be more indicated in the early stages of this disease, where inflammation predominates. In the same line, Giombini et al. (2016) published a randomized control trial comparing three kinds of injections: medical ozone, hyaluronic acid, and the combination. This last group showed the best outcomes and the hyaluronic acid group was more effective than ozone but without statistically significant differences. A systematic review and meta-analysis from Li et al. (2018) comparing medical ozone and hyaluronic acid had similar results, checking that hyaluronic acid was better than ozone but both improved the clinical status of the patients and had no significant side effects. A publication from Lopes de Jesus et al. (2017) demonstrated through a multicenter randomized, double-blind clinical trial the efficacy of intra-articular ozone injections versus placebo in knee osteoarthritis. In 2018 (Anzolin and Bertol, 2018; Costa et al., 2018; Raeissadat et al., 2018), 2019 (Arias-Vázquez et al., 2019; Noori-Zadeh et al., 2019) and 2020 (Sconza et al., 2020), six systematic reviews and meta-analyses were published in different journals supporting the use of ozone in knee osteoarthritis with a high level of evidence (GRADE 1+; Table 1).

**Table 1:** Systematic reviews and meta-analysis on medical ozone in knee osteoarthritis (Anzolin and Bertol, 2018; Costa et al., 2018; Raeissadat et al., 2018; Arias-Vázquez et al., 2019; Noori-Zadeh et al., 2019; Sconza et al., 2020).

**Author (year)**	**Study type**	**Number of studies**	**Route of O**_**3**_ **application**	**Sample size**	**Findings**
Anzolin and Bertol, 2018	Systematic review	9: 7 RCTs 1 prospective non-controlled 1 experimental pre-clinical	8: Intra-articular gas injection 1: Rectal inssuflation	2,175 patients 180 mice	In 7 studies, ozone was better that placebo or othr drugs. In 2 studies, ozone was better than placebo but worse than other drugs. No reference to side effects
Costa et al., 2018	Systematic review	6: Not detailed	Intra-articular gas injection	Uknown	There is no clear recommendation for ozone treatment. Only one^*^ moderate quality RCT. Ozone was better than placebo but similar or worse than other drugs or technique. No severe adverse reactions
Raeissadat et al., 2018 ^**^	Systematic review Meta-analysis	5 RCTs	Intra-articular gas injection	428 patients	Ozone was significantly superior to placebo and slightly lower to other control injections with non-significant difference. Only one^*^ moderate quality RCT Side affects rate similar to control group. No severe adverse reactions observed
Arias-Vázquez et al., 2019 ^**^	Systematic review	10 RCTs	Intra-articular gas injection	781 patients	Ozone was significantly superior to placebo and similar to other control injections with non-significant difference. Only two^*^ moderate quality RCT Side affects rate similar to control group. No severe adverse reactions observed
Noori-Zadeh et al., 2019 ^**^	Systematic review Meta-analysis	10 RCTs	Intra-articular gas injection	419 patients	Ozone was significantly superior to placebo and similar to other control injections with non-significant difference Only two^*^ moderate quality RCT No reference to side effects
Sconza et al., 2020 ^**^	Systematic review	11 RCTs	Intra-articular gas injection	858 patients	Ozone was significantly superior to placebo and similar to other control injections with non-significant difference Only two^*^ moderate quality RCT No severe adverse reactions observed

RCT, randomized control trial.

* RCT 1+ according GRADE classification (https://www.gradeworkinggroup.org/).

** Moderate quality systematic review/meta-analysis according SIGN cheklist (https://www.sign.ac.uk/what-we-do/methodology/checklists/).

These studies do not compare the results according to the radiological status of the knee, and we think this is basic fact, because hyaluronic acid is not indicated for moderate or severe knee osteoarthritis. Further studies in this line are needed to clarify the indications for medical ozone. Cost-efficacy studies are also needed as medical ozone is far cheaper than hyaluronic acid.

Other inflammatory knee pathologies have also been studied. Peritendinosus injected ozone has been used with success in refractory knee tendinopathies (Gjonovich et al., 2003). They improved 36 athletes with “jumper’s knee” who had not improved after conventional treatments.

Patellofemoral chondromalacia is a painful pathology whose treatment is mainly surgical, after which there is often aftermath. Manzi and Raimondi (2002) treated 60 patients with injected O_2_/O_3_ that had persistent pain after conventional surgical treatment, obtaining a higher and faster resolution of the pain in the treatment group compared to the control group.

### Ozone Therapy in Shoulder Pathology

Gjonovich et al. (2002) published a prospective controlled study of patients suffering from subacromial tendinopathy treated with injected ozone versus mesotherapy; the ozone group showed superior clinical results. Ikonomidis et al. (2002) demonstrated in a double-blind clinical trial the greater efficacy of injected O_2_/O_3_ versus steroid injections and physiotherapy also in subacromial tendinopathy.

Oxygen-ozone therapy has also been used successfully in combination with shock waves, to treat calcifying tendinitis of the shoulder (Trenti and Gheza, 2002). Brina and Villani (2004) have published the utility of ultrasound-guided O_2_/O_3_ infiltrations in patients with non-surgical lesions of the rotator cuff. Medical ozone can increase the efficacy of other substances, as it was shown in paper of Moretti (2011) with hyaluronic acid and in paper of Gurger et al. (2021) with platelet-rich plasma (PRP); this last paper verifies the collaborative effect of ozone and PRP from a histological point of view.

### Ozone Therapy in Spinal Pathology

Undoubtedly, the largest number of published works focuses on the use of ozone therapy for the treatment of herniated disks, both cervical and lumbar.

The treatment of cervical herniated disks is generally more conservative than that of the lumbar, perhaps due to the higher index of serious complications from your surgery (Wang et al., 2007). In this context, the interest in intradiscal or paravertebral medical ozone injections has special relevance, and the analgesic, anti-inflammatory, and muscle relaxant effects of the ozone therapy in cervical pathology have been described (Albertini, 2002; Villa, 2002).

In 2004, Moretti et al. (2004b), conducted a clinical trial comparing the efficacy of ozone therapy versus mesotherapy in patients with neck pain, upper limb paresthesias (uni or bilateral), peripheral vertigo, and headache. One hundred and fifty two patients had hernias, protrusions, or spondylosis, 76 of which were treated with medical ozone infiltrations in paravertebral muscles, the trapezius, and the elevators of the scapula; the other 76 patients were treated with anti-inflammatory drugs and mesotherapy. The differences were statistically significant in favor of the group treated with oxygen-ozone with 78% of optimal or good results, compared to 56.25% in the group of mesotherapy.

Regarding cervical intradiscal injections, a work published by Xiao et al. (2006) also supports their efficacy. Eighty-six patients with cervical spondylosis treated with CT-guided infiltrations were retrospectively assessed. Thirty-seven suffered from myelopathy, 30 had radiculopathy and 19 had sympathetic symptoms. The indication for treatment was cervicalgia with neurological symptoms (brachial irradiation patterns, loss of tenderness, tingling, numbness, muscle weakness, or deficiency of deep tendon reflexes) and all should be refractory to conservative therapies for at least 12 weeks. Patients with cervical spinal stenosis, ossification of the posterior longitudinal ligament were excluded. The treatment with ozone therapy was excellent, good, or poor in 78, 16, and 6% of the cases, respectively, as recorded with the modified MacNab Scale. These results coincide with those published by Alexandre et al. (2005) in the same year.

In lumbar pathology the number of published works is large. The good results, along with the safety of the technique and the dreaded complications of the surgery, have made more and more authors consider ozone therapy, either paravertebral or intradiscal, the first choice in case of failure of conservative treatment.

Andreula et al. (2003) added intradiscal and periganglionic O_2_/O_3_ to the infiltration with local anesthetics and steroids; in the 6 months follow-up, by blind evaluators, a statistically significant improvement was observed with the combination of both treatments. No side effect was detected. Buric et al. (2003) did a prospective follow-up over 18 months of 104 patients with lumbosciatic pain due to disk protrusions treated with intradiscal and intraforaminal O_2_/O_3_ injections, finding improvements in pain and functional capacity in most of them. Disk volume measurements were made with MRI and it was observed that, at 5 months, 22% of the protrusions had not changed in volume, 41% had reduced the size and 37% had disappeared. The results indicated that the technique was effective in the treatment of protrusions, although the efficacy was not higher than that of the microdiscectomy, according to the authors’ experience (Buric, 2005). Also regarding disk protrusions, Qing et al. (2005), in a sample of 602 patients and 1,078 operated disks, concluded the suitability treatment with ozone therapy as the first choice after failure more conservative techniques. When comparing ozone therapy with other minimally invasive techniques, these authors considered that it was an effective, safe, and minimally stressful technique for the patient and easy to perform.

Bonetti et al. (2005), in a randomized clinical trial, compared the efficacy of intraforaminal infiltration of O_2_/O_3_ with periradicular infiltration of steroids. Three hundred and six patients with low back pain and neuropathic leg pain with and without disk disease (spondylosis, spondylolisthesis, and facet joint degeneration) were recruited and randomly divided into two groups (166 and 140). The main measuring instrument was the modified MacNab scale. Patients were evaluated in the short (1 week), medium (3 months), and long term (6 months). In short term, there were no statistically significant differences between the two treatment modalities (*p* = 0.4077). In long term, the differences in favor of ozone treatments were statistically significant, but only in the group of patients with disk disease (*p* = 0.0021); also in long term, it could be seen that ozone therapy treatments had statistically lower failure rate (8.6%) than treatments with steroids (21.4%).

Muto et al. (2008) performed CT-guided injections to 2,900 patients with lumbosciatic pain due to herniated disks (including relapses). The gas was injected intradiscal, peri-ganglionic, and periradicular. After a month the patients were reviewed, repeating the session in those cases in which the improvement was partial. At 6 and 12 months, there were improvements of 75–80% with simple disk herniation, 70% with multiple hernias, and 55% with relapses.

Equally positive results are obtained by Castro et al. (2009), in a prospective observational study in which they treated 41 patients with simultaneous intradiscal, epidural, and periradicular infiltrations. Patients with a herniated disk and free fragment and/or major neurological deficit were excluded. The evolution was very positive (according to the VAS and the Laitinen test) from the first to the last of the post-basal records (at 30 days and 6 months, respectively), and the degree of satisfaction was rated as good by 85.4% of the sample.

To clarify the long term results of this technique, Torres et al. (2009) designed a study in patients with sciatica due to herniated disc by applying three consecutive sessions of O_2_/O_3_ infiltrations once a week; the first two with an epidural [adding bupivacaine (5 ml to 0.25%) and triamcinolone (4 mg)] and paravertebral ozone and the third one, only with intradiscal ozone. The evolution of 91 patients was recorded for 24 months. A very significant improvement was achieved in 95.6% of the patients at the first-month follow-up visit, 87.7% at the first-year visit, and persisted at the end of the follow-up in 84.1% of the sample. They got one case of discitis and 11 cases of temporary headache and four cases of temporary low back pain. They found a significant reduction of the size of the hernia in 79% of the patients at 24 months MRI.

Although most of the works published refer to intradiscal injections, paravertebral injections are safer, more simple, and the most used in clinical practice, although their efficacy was under discussion. In 2006, a randomized clinical trial was published (Zambello et al., 2006) comparing the efficacy of muscular paravertebral infiltration of O_2_/O_3_ with that of epidural steroids in patients refractory to conventional treatment (oral steroids and muscle relaxants). One hundred and seventy one patients were treated with epidural steroids and 180 underwent paravertebral infiltrations of medical ozone. At the 3 weeks follow-up, the improvement was statistically significant in favor of patients treated with ozone therapy (total or almost total remission of pain in 88.2%, compared to 59% in the steroids group), and at 6 months the evolution was excellent or good in 77.1% of patients treated with ozone therapy, compared to 47.3% of patients treated with steroids.

A double-blind randomized clinical trial was conducted (Paoloni et al., 2009) to better assess the efficacy of paravertebral ozone infiltrations in the treatment of acute low back pain due to herniated disk. Sixty patients were recruited and randomized into two groups; one was treated with real infiltrations and in the other group these were simulated. Follow-up was done 15, 30, 90, and 180 days after ending the treatment. Patients treated with true ozone injections significantly improved the pain and functional limitation (*p* < 0.05), requiring less analgesic medication.

In 2010, a meta-analysis was published (Steppan et al., 2010) on the efficacy and safety of ozone therapy for the treatment of herniated disks. Twelve studies were included with a total sample of 8,000 patients; the mean improvements recorded were similar to those reported for discectomy: 3.9 points out of 10 on the visual analog pain scale, 25.7 points in functional capacity according to the Oswestry Disability Index and a 79.7% improvement in the records of the modified MacNab scale. The percentage of complications was 0.064%, so the treatment was considered safe and effective. Two years later, Magalhaes et al. (2012) published a systematic review and meta-analysis compiling eight observational studies and four randomized clinical trials. They concluded that the intradiscal injection to treat lumbar disk herniation has a recommendation level of 1C and that the paravertebral treatment has a recommendation level 1B according to the criteria of the US Preventive Services Task Force; this means that the recommendation is strong (maximum level), although with certain reservations for intradiscal injection due to the diversity of existing protocols.

About 80% of the population in Western countries will experience at least one episode of low back pain in your lifetime, and 55% of these will have an associated radicular pain (Long, 1991). Failed back surgery syndrome ranges between 15% and 20%, which leads to propose more conservative and less invasive treatments, such as ozone therapy, whose effectiveness seems to oscillate between 65 and 80% suggesting that a small change in disk volume can produce a large clinical change (Gangi et al., 1998). Complications of open surgery must also be taken into account, such as fibrosis, epidural and perineural tears, nerve adhesions, limitations of biomechanics due to fibrosis, and muscle paravertebral spasms and associated myofascial syndromes (Manchikanti et al., 2007). In this context, infiltrations with O_2_/O_3_, both at the paravertebral level and trigger points of related musculature and percutaneous intradiscal injection with ozone, are techniques on the rise due to their efficacy, ease of execution, low cost, and very few important side effects.

Andreula et al. (2003), when comparing ozone intradiscal injection with enzymatic nucleolysis, conclude that, with similar clinical results, treatment with ozone therapy would be the first choice due to its advantages like the ones that follow:

- There is no possibility of allergic reactions or anaphylactic
- Possibility of repeating the treatment as many times as it is considered
- Theoretical lower risk of infections, due to the germicides properties of ozone
- Possibility of using a thinner needle due to ozone fluidity and, therefore, a so much less traumatic injection
- Less post-infiltration discomfort (2, 3 days vs. 1 or 2 weeks).

To these advantages would be added those described over corticosteroids due to the absence of ozone adverse effects. In this regard, Fernández et al. (1998), in their review on the use of corticosteroids draw attention to the following points:

- Infectious arthritis
- Progressive joint deterioration
- Soft-tissue atrophy and hypopigmentation
- Tendon rupture
- Reactive synovitis due to glucocorticoid microcrystals
- Systemic adverse effects.

Some recent publications remark that the ozone administration does not close the path to surgery in case of failure (Bocci et al., 2015; Muto et al., 2016).

Finally, it is worth mentioning that the efficacy of ozone therapy in the treatment of failed back surgery syndrome, highly prevalent among spine-operated patients, and usually worsens with new surgeries. In these patients, we observe fibrosis due to epidural and perineural scars, paravertebral spasms, and neural adhesions, whose chronic inflammatory stimulus would lead to neuroplastic phenomena with central and peripheral sensitization. Theoretically, the fibrinolytic, anti-inflammatory and antioxidant properties of the infiltrated O_2_/O_3_ would make it ideal for the treatment of these processes. The team from the National Medical Center 20 de Noviembre, in Mexico City, has published two papers treating 30 patients in each work. On both studies applied a first epidural injection together paravertebral infiltration followed by three weekly sessions of paravertebral infiltrations; doses of 20 ml at 30 μg/ml in the first paper and the same amount at 50 μg/ml in the second one, but the treatments could not improve patients’ pain (Grijalva et al., 2012; Hernández et al., 2012). A promising alternative would be the combination of repeated paravertebral injections combined with epidural ones. Padilla del Rey et al. (2015) reported very significant improvement (VAS of 10 a 3) in a patient with refractory post-laminectomy pain who received three epidural infiltrations with ozone (10 ml at 20 μg/ml concentration) in three consecutive weeks and six paravertebral infiltrations, twice a week at the same time (10 ml at 10–30 μg/ml progressive concentration). Anyway, given the scale of the problem, more studies are needed.

### Infiltrative Ozone Therapy in Rheumatoid Arthritis

A preclinical study carried out in Nanfang Hospital compared the effects of medical ozone infiltrations at different concentrations compared to oxygen; the authors showed that intra-articular ozone injected at a concentration of 40 μg/ml is capable of inhibiting synovitis in rats with rheumatoid arthritis (Chen et al., 2013). Some doctors use ozone therapy empirically in patients with rheumatic diseases using joint infiltrations for years, supposedly with very positive results, but there are no major works published in this regard.

### Other Applications of Infiltrated Oxygen/Ozone

Other indications published in case series studies are tendinopathies and neural entrapment syndromes, lateral epicondylitis, rhizoarthrosis, spondylolisthesis, spondylolysis, plantar fasciitis, septic spondylodiscitis, D′Quervain’s tenosynovitis, metatarsalgia due to postsurgical fibrosis after resection of Morton’s neuroma or temporomandibular joint pathology (Bonetti et al., 2002, 2011; Gheza, 2002; Gheza et al., 2002; Zanardi and Zorandi, 2002; Bonetti, 2003; Gaffuri et al., 2003; Ikonomides et al., 2003; Moretti et al., 2005; Alvarado, 2006; Babaei-Ghazani et al., 2019).

## Systemic Ozone Therapy in Pain Medicine

As mentioned, ozone therapy would be indicated as an adjuvant treatment of diseases related to disturbance of cellular redox balance or tissue oxygenation. From this point of view, systemic ozone therapy would help patients with pain chronic, as recent preclinical studies have demonstrated the role of ROS in hyperalgesia, *via* activation of the N-methyl-D-aspartate (NMDA) receptors. Gao et al. (2007), in a preclinical model of pain, both neuropathic and inflammatory, could demonstrate that ROS were increased at the dorsal horn in these patients, and that systemic administration of a neutralizing agent of ROS reduced the hyperalgesia by blocking phosphorylation from NMDAs. Later, the same research group (inducing hyperalgesia by capsaicin in rats) was able to demonstrate the role of the superoxide anion as responsible for abnormal pain signal processing in the dorsal horn, suggesting the therapeutic role of mitochondrial SOD-2 in these types of pain (Schwartz et al., 2009).

But certainly, the levels of scientific evidence in the treatment with systemic ozone therapy for chronic pain are practically non-existent. In this regard, a study by Clavo et al. (2013), on chronic headache refractory to triptans, found that systemic ozone improved pain and frequency of crises. Also, Fernández et al. (2016) published a work in which the use of systemic ozone therapy improved efficacy and decreased the side effects of methotrexate in patients with rheumatoid arthritis. Theoretically, the beneficial effect on immunosuppressed patients would make it useful, both in the treatment of herpes zoster infection and postherpetic neuralgia (Hu et al., 2018). In this field, several experts have tried ozone treatments for years, apparently with positive results. Ozone therapy is generally used as an adjunct to conventional treatments, either systemic or local (infiltrations or applications of oils and ozonated water; Mattassi et al., 1985; Delgado et al., 1989; Konrad, 2001). These studies have a low evidence level but are useful to encourage the development of better-designed works.

Fibromyalgia seems to be a “stress disease” in which underlies an alteration of the cellular redox balance, consequence of an increase in the production of free radicals, a deficiency of organic antioxidant capacity, or both simultaneously. Biochemical findings support this fact and systemic ozone therapy has been proposed (Borrelli and Bocci, 2002; Hidalgo, 2012). Hidalgo et al. (2005) treated 21 patients with fibromyalgia refractory to a multidisciplinary treatment with 10 sessions of autohemotherapy, finding very good tolerability and improvement in pain and fatigue, as well as a significant decrease in the use of pain medication. This same research group (Hidalgo-Tallón et al., 2013) treated 36 patients with fibromyalgia with one daily dose of rectal ozone (200 ml of gas at 40 μg/ml) during 24 sessions, reporting significant improvements in the Fibromyalgia Impact Questionnaire, the state of mind, and the physical component of the quality of life using SF-12 scale. The treatment was very well tolerated, with transient meteorism as the most relevant adverse effect.

Systemic ozone has also been tested together with infiltrations in patients with rheumatoid arthritis. A Calunga et al. (2007) research group, published a study about lumbar disk herniation comparing paravertebral infiltrations isolated or combined with rectal ozone and checked that the combination improved all clinical parameters. In 2010, they compared (Méndez-Pérez et al., 2010) isolated O_2_/O_3_ intra-articular infiltrations (3 ml at 10 μg/ml) with the infiltrations plus rectal ozone in patients with rheumatoid arthritis of the temporomandibular joint; improvements, both in pain and in function and state of the joint capsule were statistically significant in favor of the combination therapy. In this regard, systemic ozone therapy seems to decrease in these patients interleukin 1 beta levels, directly related to disease activity, while intraarticular ozone injections would decrease intraarticular interleukin 8 levels, justifying lower granulocyte count and clinical improvement (Eastgate et al., 1988; Menéndez et al., 1989; Fahmy, 1993, 1995).

## Safety and Contraindications of Ozone Therapy

All authors agree on the high safety of treatments with ozone therapy, especially modern medical ozone generators with great precision.

Jacobs (1982) published that the incidence of effects adverse effects of systemic ozone therapy was only 0.0007%, mainly nausea, headache, and fatigue. In Cuba, with 25 years of experience, having at least one ozone therapy unit per province of the country, only slight adverse effects have been recorded (Bocci, 2002).

The experience of Italian experts is similar, although Dr. Bocci (Ajamieh et al., 2005) describes at least six deaths from gas applications in a direct intravenous way, a practice not recommended by scientific associations (Baeza et al., 2015).

Eventually, the most serious common adverse effect would be a vagal reaction, generally associated with pain during infiltration. It is necessary to note that the injection must be done slowly, especially if a large gas volume at a high concentration is used (Zambello et al., 2004).

However, some other complications have been reported in the literature, most of them due to malpractice or without a causal relationship between the ozone administration and the adverse event (Marchetti and La Monaca, 2000; Corea et al., 2004; Lo Giudice et al., 2004; Ginanneschi et al., 2006; Gazzeri et al., 2007; Bo et al., 2009; Seyman et al., 2012; Vilas-Rolan et al., 2012; Fort et al., 2014; Menéndez et al., 2014; Mustafa et al., 2015; Vanni et al., 2015; Cano et al., 2016; Salvatore et al., 2016; Balan et al., 2017; Hüseyin et al., 2017; Wen-Juan et al., 2017; Beyaz et al., 2018; Yang et al., 2018; He et al., 2019). Most of them disappeared in a few days not needing specific medical treatment.

An absolute contraindication is severe glucose-6 deficiency phosphate dehydrogenase (favism), as this enzyme is necessary for supply hydrogen ions to the glutathione system, responsible for buffering the oxidation that lipoperoxides will produce in red blood cells (Viebahn, 2007).

As relative contraindications to systemic ozone therapy would be uncontrolled hyperthyroidism, thrombocytopenia, severe cardiovascular instability, and seizure states. It is also not advisable to use systemic ozone therapy in pregnant patients (Bocci, 2002) as it has not been deeply tested. Infiltrations should be avoided following the general criteria described in the literature (Sánchez et al., 2006).

Undoubtedly, ozone therapy should be practiced by a well-trained doctor and, in patients with a poor general condition, a diagnosis of the pro-oxidant/antioxidant status of the patient would be advisable.

## Conclusion

For knee osteoarthritis and lumbar disk herniation, evidence on safety and efficacy from systematic reviews and meta-analysis, according the quality of the evidences proposed by the Scottish Intercollegiate Guidelines Network (SIGN), including GRADE criteria, are high (1+ and 2+ studies) and allow a recommendation level B, the same observed in most of the techniques used presently in pain units (SIGN, 2019).

However, for the rest of potential indications, the evidence level is low and ozone must be used only when other conventional treatments have failed or in a compassionate way.

Lack of standardization in treatment protocols is the Achilles’ heel of this technique, probably because of his great tolerablity that encourage doctors to explore different dosages without comparing efficacy between them. This is the main criticism in systematic reviews’ conclusions.

More RCT are needed to increase our knowledge on the indications of medical ozone, but safety profile is good enough so as to develop them. Fortunately, research interest on ozone therapy is growing, as we can check by simply doing a basic research in PubMed and watching the evolution of the number of publications in the past 20 years.

## Author Contributions

All authors listed have made a substantial, direct, and intellectual contribution to the work, and approved it for publication.

## Conflict of Interest

The authors declare that the research was conducted in the absence of any commercial or financial relationships that could be construed as a potential conflict of interest.

## Publisher’s Note

All claims expressed in this article are solely those of the authors and do not necessarily represent those of their affiliated organizations, or those of the publisher, the editors and the reviewers. Any product that may be evaluated in this article, or claim that may be made by its manufacturer, is not guaranteed or endorsed by the publisher.
